# A case of alpha-fetoprotein-positive thymic small cell carcinoma: a case report

**DOI:** 10.1186/s40792-023-01750-4

**Published:** 2023-09-18

**Authors:** Masao Kobayashi, Soichiro Funaki, Eriko Fukui, Eiichi Morii, Yasushi Shintani

**Affiliations:** 1 Department of General Thoracic Surgery, Osaka University Graduate School of Medicine, 2-2 L5, Suita city, Osaka 565-0871, Japan; 2 Department of Pathology, Osaka University Graduate School of Medicine, 2-2, Suita city, Osaka 565-0871 Japan

**Keywords:** Thymic carcinoma, Neuroendocrine tumor of thymus, Alpha-fetoprotein-producing thymic small cell carcinoma, Germ cell tumor

## Abstract

**Background:**

An alpha-fetoprotein (AFP)-positive neuroendocrine tumor of the thymus is a rare thoracic malignancy. Few cases of AFP-positive thymic large cell neuroendocrine carcinoma have been reported, with no known previous report of an AFP-positive thymic small cell carcinoma. We encountered a patient with an AFP-positive small cell carcinoma and report here the clinical course.

**Case presentation:**

A 40-year-old man was transferred to our hospital for a large anterior mediastinal tumor and showed an elevated serum AFP level. Computed tomography-guided biopsy results led to diagnosis of small cell carcinoma. Induction chemoradiotherapy was performed before surgery because of pulmonary artery invasion. The response to Induction chemoradiotherapy varied among sites, with the main tumor showing shrinkage and the metastasis site growth. This discrepancy suggested a histologic type unresponsive to or cancer cells potentially resistant to chemotherapy, thus a surgical re-biopsy was performed and histological findings revealed AFP-positive small cell carcinoma. Additional chemotherapy was performed, though could not control cancer progression, and the patient died 8 months after the first medical examination.

**Conclusions:**

Our present clinical experience indicates the importance of histological examination for determining AFP-positive anterior mediastinal tumor treatment. Although AFP-positive neuroendocrine tumor of the thymus is relatively rarer than germ cell carcinoma, differential diagnosis with use of a histological examination should be considered because of the potentially poorer prognosis. The present clinical findings for an AFP-positive neuroendocrine tumor of the thymus case are considered useful for establishing an optimal treatment strategy in the future.

## Background

A neuroendocrine tumor of the thymus (NETT) has been included in the World Health Organization classification since 2015 [1]. NETTs are classified into four groups according to histological features, typical and atypical carcinoids, large cell neuroendocrine carcinoma (LCNEC), and small cell carcinoma. LCNEC and small cell carcinoma are poorly differentiated types of carcinomas classified as high-grade, and affected patients generally have a poor prognosis. A NETT is rare and accounts for approximately 4–7% of anterior mediastinal tumors [2], while a thymic small cell carcinoma is an even more rare thoracic malignancy, comprising only about 10% of NETT cases [3]. To the best of our knowledge, few cases of AFP-positive thymic LCNEC have been reported, with no known previous report of an AFP-positive thymic small cell carcinoma. We encountered a patient with an AFP-positive small cell carcinoma and report here the clinical course.

## Case presentation

**Fig. 1:**
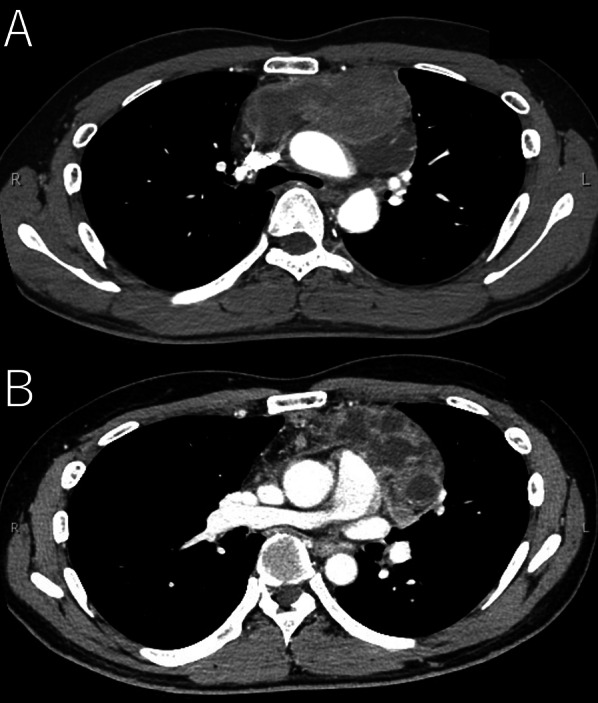
CE-CT images showing huge tumor in anterior mediastinum. **A** Compressed trachea and esophagus. **B** Suspected invasion to main trunk of pulmonary artery, without distal metastasis

A 40-year-old man visited a clinic complaining of chest tightness. Computed tomography (CT) scanning revealed a 15 × 6 × 5 cm tumor in the anterior mediastinal region and he was transferred to our hospital due to the risk of airway compression by the large tumor. Oxygen saturation level upon admission was 97% room air and other vital signs were also stable. His past medical history was insignificant, though there was a smoking history of a pack per day for 20 years. In laboratory results, there were no abnormal levels, other than elevation of the serum tumor markers alpha-fetoprotein (AFP) at 619 ng/ml and neuron-specific enolase (NSE) at 69.4 ng/ml. Chest X-ray results showed a widened mediastinum. Systemic contrast-enhanced computed tomography (CE-CT) scanning revealed a 15 × 9 × 5 cm anterior mediastinal tumor with obstruction of the trachea and esophagus (Fig. 1A), while invasion to the main trunk of a pulmonary artery was also suspected (Fig. 1B). Positron emission tomography–computed tomography (PET–CT) indicated a high accumulation of the main tumor (SUV-max 13.2), with no lymph node or distal metastasis.

**Fig. 2:**
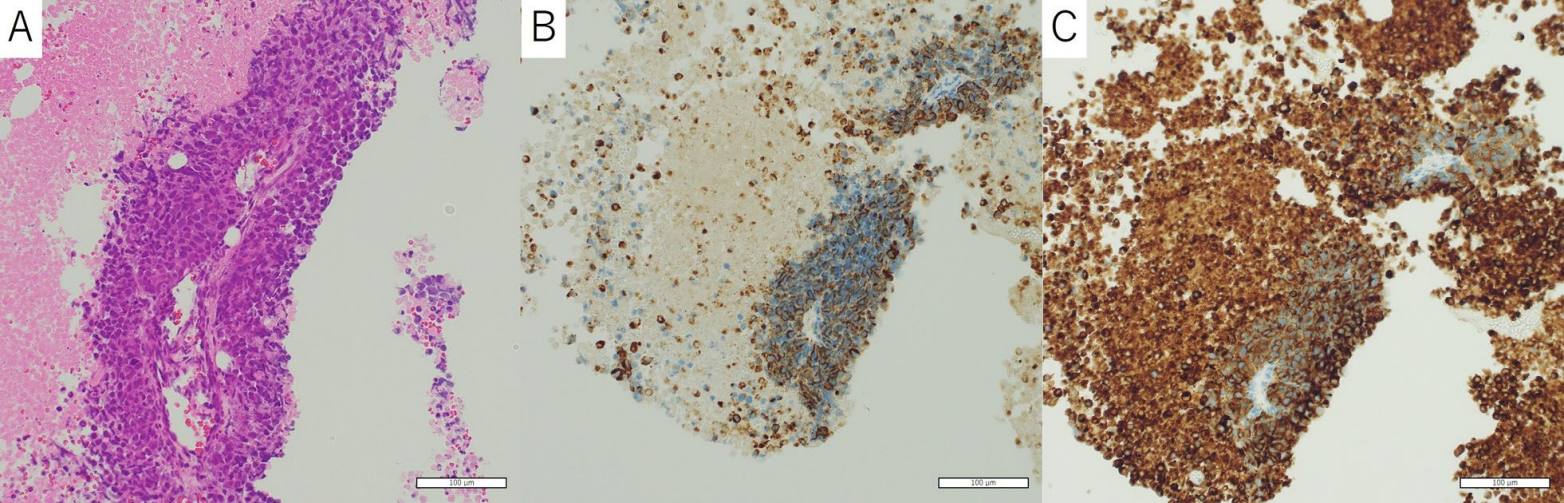
Histological findings of CT-guided biopsy specimen. **A** High nucleus to cytoplasm ratio in tumor cells. Immunohistochemical findings positive for **B** chromogranin A and **C** synaptophysin

Initially, the patient was diagnosed with a germ cell tumor based on a high serum AFP level and imaging findings, and a CT-guided biopsy from the main tumor was performed for diagnosis confirmation. However, a histopathological examination revealed a small cell carcinoma, for a high nucleus-to-cytoplasm ratio in the tumor cells (Fig. 2A), as well as immunohistochemically positive findings for chromogranin A (Fig. 2B), and synaptophysin (Fig. 2C). Based on the imaging findings, the tumor was diagnosed as clinical stage IIIB thymic small cell carcinoma (cT4N0M0, TNM staging 8th edition). Following discussion by the cancer board at our hospital, induction chemoradiotherapy followed by surgery was planned.

**Fig. 3:**
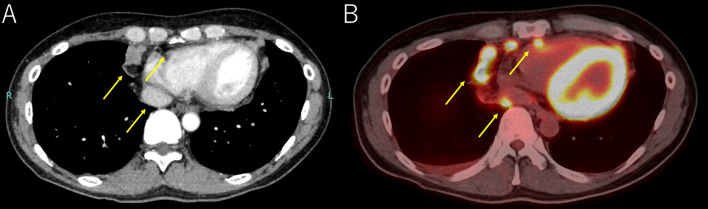
CE-CT (**A**) and PET–CT (**B**) images showing apparent bilateral pleural dissemination and para-phrenic lymph node metastasis (arrow)

**Fig. 4:**
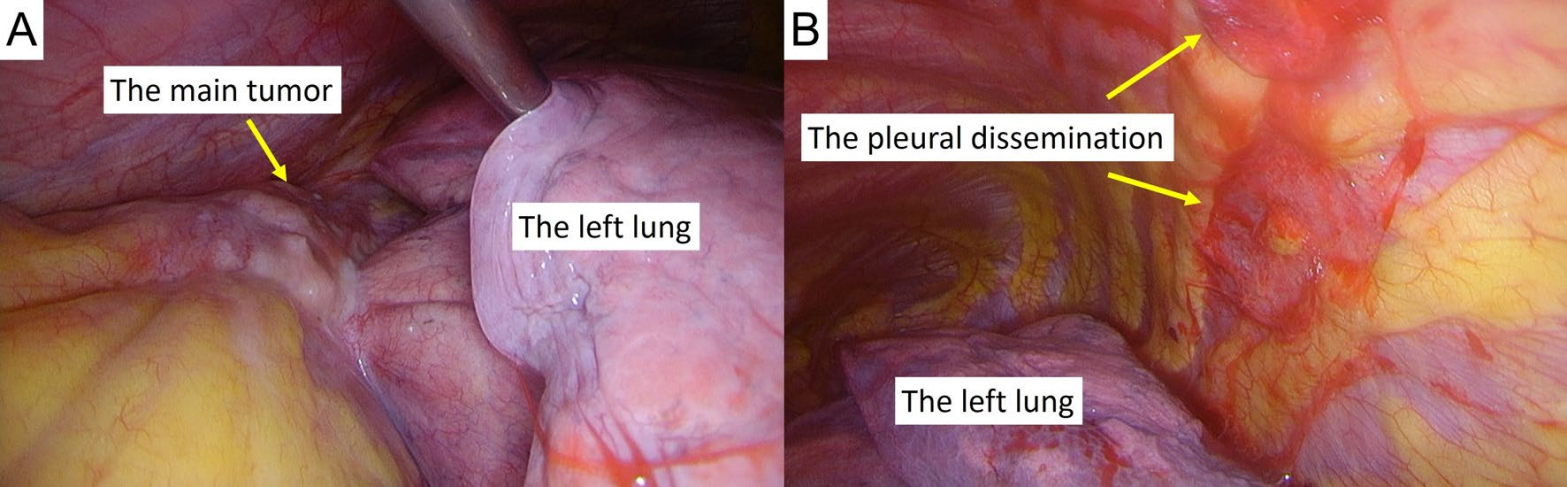
Surgical re-biopsy from both the main tumor (**A**) and pleural dissemination (**B**)

**Fig. 5:**
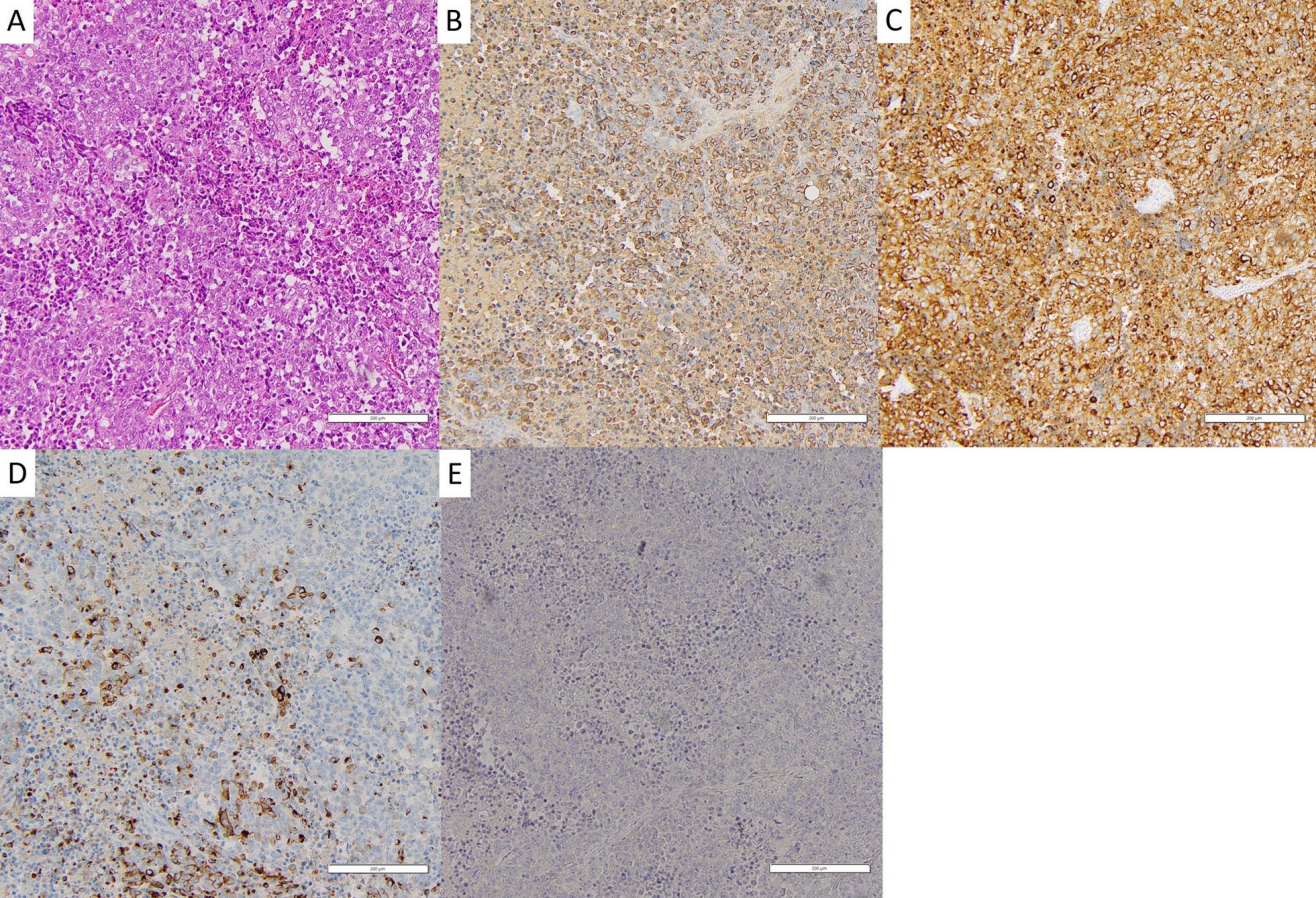
Histological findings of resected specimen. **A** High nucleus to cytoplasm ratio in tumor cells. Immunohistochemical findings positive for not only **B** chromogranin A, and **C** synaptophysin, but also **D** AFP, in contrast negative for **E** NUT

Two cycles of chemotherapy (cisplatin 80 mg/m^2^, etoposide 100 mg/m^2^ for 3 days) and radiation therapy (45 Gy/30 fractions) were administered, with partial response shown, as the tumor size was reduced to 8 × 7 × 3 cm in accord with drastically decreased serum AFP at 220 ng/ml and NSE levels at 10.8 ng/ml. A radical resection was planned for 7 weeks after radiation therapy. However, pre-operation CE-CT and PET–CT findings revealed bilateral pleural dissemination and para-phrenic lymph node metastasis (Fig. 3A, B). Because of the discrepancy in response to chemotherapy between the main lesion and site of metastasis, the possibility of a mixture with another histological type such as germ cell tumor led to a surgical re-biopsy from both the main tumor (Fig. 4A) and metastatic lesions on the left pleura (Fig. 4B). Histological examinations of the resected specimen from the both sites showed the same findings, as a high nucleus-to-cytoplasm ratio in the tumor cells (Fig. 5A), as well as immunohistochemically positive findings for chromogranin A (Fig. 5B), synaptophysin (Fig. 5C), and AFP (Fig. 5D). In contrast, neither NUT (Fig. 5E), CD 30 nor CD 117 was positive, indicating that the tumor was not NUT carcinoma or germ cell carcinoma but rather AFP-positive thymic small cell carcinoma. Following the second surgical biopsy, the patient underwent two more cycles of chemotherapy with the same regimen. Unfortunately, cancer progression could not be controlled, resulting in death 8 months after the first medical examination.

## Discussion

In the present case, a diagnosis of mediastinal tumor was considered based on occurrence site and serum tumor marker levels. In general, an anterior mediastinal tumor in an adult male with a high serum AFP level is likely to be germ cell carcinoma. Surprisingly, histological findings in our patient showed small cell carcinoma with immunohistochemical positivity for AFP, which to the best of our knowledge has never been described occurring in the thymus. A literature search found two cases of AFP-positive thymic LCNEC, both of which were histologically diagnosed based on resected specimens, though the initial clinical diagnosis was germ cell carcinoma [4, 5]. Chemotherapy is usually conducted for a germ cell carcinoma and its response rate is relatively high. On the other hand, the treatment strategy for an NETT is complete surgical resection, thus the strategies differ for these two types of tumors. Therefore, when planning treatment for cases positive for AFP in serum, histopathological analysis is especially important, because high serum AFP findings may lead to the wrong plan for treatment. It is our belief that therapy should be performed based on histological diagnosis, especially when induction chemotherapy is necessary.

The clinical course of patients with an AFP-positive neuroendocrine tumor remains to be elucidated due to the rare occurrence. Nevertheless, various originating sites have been reported, such as ovary [6], mammary gland [7], and colon [8]. Most cases were described as poor prognostic cancer because of the high likelihood of hematogenous and lymphomatous metastasis. Hence, it is conceivable that AFP-positive NETT is an unfavorable type of cancer such as seen in the present case.

There is no standard treatment for an AFP-positive NETT, though Sullivan et al. suggested that complete resection improved prognosis [9]. In this regard, advanced NETTs are thought to respond to combined modality therapy that includes chemotherapy. The regimen for the present case consisting of cisplatin and etoposide was selected based on a report by Pujol [10]. Furthermore, Takezawa et al. demonstrated that a regimen of a combination of cisplatin and paclitaxel, and BEP therapy (first-line for germ cell carcinoma) was also temporarily effective for AFP-positive NETT cases [4]. Their regimen may have positive effects, though additional studies are needed to provide a favorable prognosis for NETT patients.

## Conclusions

An AFP-positive small cell carcinoma is an uncommon tumor with no standard treatment strategy. When diagnosing an anterior mediastinal tumor with a high AFP level, germ cell carcinoma is the most common. Although AFP-positive NETT is rarer, differential diagnosis with use of a histological examination should be considered because of the potentially poor prognosis. The present clinical findings for an AFP-positive NETT case are considered useful for establishing an optimal treatment strategy in the future.

## Data Availability

The authors declare that all the data are available within this article.

## Abbreviations

NETT: Neuroendocrine tumor of the thorax
LCNEC: Large cell neuroendocrine carcinoma
CT: Computed tomography
AFP: Alpha-fetoprotein
CE-CT: Contrast-enhanced computed tomography
PET–CT: Positron emission tomography–computed tomography
NSE: Neuron-specific enolase
